# Selenium Metabolizing Capabilities of 12 Bacterial Strains Isolated from Urban Environmental Samples

**DOI:** 10.3390/microorganisms13071675

**Published:** 2025-07-16

**Authors:** Masashi Kuroda, Iori Ishimoto, Chisato Kameoka, Toshiki Kawanishi, Ren Saito, Hajime Toki, Hiroya Yamagishi, Yuzuki Watanabe, Yukinori Tani

**Affiliations:** 1 Faculty of Social and Environmental Studies, Tokoha University, 6-1 Yayoi-cho, Shizuoka 422-8581, Japan; 2 Department of Environmental Health Sciences, Graduate School of Nutritional and Environmental Sciences, University of Shizuoka, 52-1 Yada, Shizuoka 422-8526, Japan; taniy@u-shizuoka-ken.ac.jp

**Keywords:** selenium metabolism, biogeochemical cycle of selenium, *Citrobacter* spp.

## Abstract

The role of bacterial selenium metabolism in non-polluted environments remains underexplored within the selenium biogeochemical cycle. In this study, selenium-metabolizing bacteria were isolated from urban environmental samples. Among 12 isolates, 10 were identified as *Citrobacter* spp., while the remaining 2 were *Scandinavium hiltneri* and *Klebsiella aerogenes*. The *Citrobacter* isolates demonstrated high selenium-removal efficiency, removing over 95% of 5 mM selenium from the aqueous phase within one week. In contrast, *S. hiltneri* K24-1 and *K. aerogenes* K24-4 removed only 19% and 69%, respectively. A detailed investigation of five representative isolates, *C. freundii* K21-1, *S. hiltneri* K24-1, *C. braakii* K24-2, *K. aerogenes* K24-4, and *C. freundii* K24-5, revealed that *Citrobacter* spp. efficiently reduced selenate directly to elemental selenium, with minimal accumulation of selenite intermediates. These results highlight *Citrobacter* spp. as key selenium reducers and suggest their potential as bioindicators of selenium metabolic capacity in the environment.

## 1. Introduction

Selenium (Se), a metalloid element with atomic number 34, belongs to Group 16 (the chalcogens) of the periodic table and exhibits chemical properties similar to those of sulfur [1]. Widely distributed in the Earth’s crust, selenium enters surface waters through both natural processes, such as volcanic activity, soil erosion, and forest fires, and anthropogenic activities, including mining, fossil fuel combustion, agricultural irrigation, and the discharge of wastewater and sewage sludge [2]. In industrial contexts, selenium is primarily recovered as a by-product of electrolytic copper refinement. It is utilized in the production of solar panels, as an additive in glass manufacturing, and as a dietary supplement for both humans and livestock [3]. These uses contribute to Se release into the environment, raising concerns due to its dual role as an essential micronutrient and a potential toxicant. Se is required by mammals in trace amounts, largely due to its incorporation into selenoproteins such as formate dehydrogenase. However, excessive Se exposure can result in adverse health effects, necessitating the regulation of its concentration in aquatic ecosystems [1].

Microbial processes are recognized as key drivers in the environmental transformation and mobility of Se [4]. Under oxidizing conditions, Se predominantly exists as the oxyanions selenate (SeO_4_^2−^; Se(VI)) and selenite (SeO_3_^2−^; Se(IV)). Microorganisms capable of Se metabolism can reduce these soluble species to insoluble elemental Se (Se^0^) or transform them into volatile methylated compounds (e.g., dimethyl selenide [CH_3_SeCH_3_], dimethyl diselenide [CH_3_SeSeCH_3_]), significantly altering Se’s environmental fate and bioavailability [5].

Numerous Se-metabolizing bacteria have been isolated and studied to elucidate their metabolic pathways [6,7,8,9,10,11,12]. *Thauera selenatis*, for example, was isolated from a bioreactor treating Se-laden wastewater [6]. It reduces Se(VI) to Se(IV) via a periplasmic Se(VI) reductase complex (SerABC) [7,8], followed by non-specific reduction of Se(IV) to Se^0^ via nitrite reductase [9]. This organism is a foundational model for understanding Se respiration at the molecular level. Other notable strains include *Enterobacter cloacae* SLD1a-1, isolated from Se-contaminated waters in California’s San Joaquin Valley [10], and *Pseudomonas stutzeri* (the bisionym of *Stutzerimonas stutzeri* [11]) NT-I, recovered from an Se smelting facility’s wastewater treatment plant [12]. The latter not only performs reductive transformations but also produces methylated Se compounds under aerobic conditions [13]. In recent years, culture-independent approaches have been employed to investigate selenium-metabolizing bacteria. Sarkar et al. (2025) analyzed selenium oxyanion-reducing microbial communities in flue-gas desulfurization wastewater through 16S rRNA gene sequencing and metagenomic analysis and inferred the involvement of *Anaerosolibacter* sp., *Mesobacillus* sp., and *Tepidibacillus* sp. in selenium oxyanion reduction [14]. While the majority of Se-metabolizing bacteria have been investigated in specific environments heavily polluted with Se or other toxic elements, knowledge about Se-metabolizing bacteria inhabiting urban environments that are potentially subject to future pollution from anthropogenic activities remains limited. Therefore, to gain a comprehensive understanding of Se’s biogeochemical cycle and the role of microbial communities in regulating its dynamics, it is essential to study Se-metabolizing bacteria from urban environments.

This study aims to isolate and characterize Se-metabolizing bacteria from urban environments in Japan. Environmental samples, including aquatic plants from home gardens, urban drainage ditches, and rivers, were collected from 12 distinct sites. Novel Se-metabolizing strains were successfully isolated, and representative bacteria were further analyzed to determine temporal changes in Se speciation. These findings offer new insights into Se cycling in typical urban freshwater ecosystems and expand our understanding of the ecological roles of Se-transforming bacteria.

## 2. Materials and Methods

### 2.1. Growth Medium

Bacto^TM^ Trypticase Soy Broth (TSB; Becton–Dickinson, Franklin Lakes, NJ, USA) or low-concentration TSB with inorganic salts (TSB-MS) was used for the enrichment and the cultivation of Se-metabolizing microorganisms. The composition of TSB-MS is as follows: 1 g/L TSB, 1 g/L (NH_4_)_2_SO_4_, 0.5 g/L Sodium Citrate · 2H_2_O, 0.1 g/L MgSO_4_ · 7H_2_O, 3.24 g/L Sodium Acetate (pH7.0), 10 mM HEPES (pH7.0), 0.14 g/L K_2_HPO_4_, and 0.04 g/L KH_2_PO_4_. Both media were sterilized by autoclave (121 °C, 20 min), and sodium selenate was added to a final concentration of 5 mM as needed. For static and aerobic cultivation, 20 mL of medium was added to a 50 mL vial and capped with a silicone sponge plug. The anaerobic culture medium was prepared by replacing the gas phase of the vial with nitrogen (99.99%) and then sealing it with a butyl rubber septum and an aluminum cap. The TSB and TSB-MS agar media were prepared by adding 20 g/L of Quality Agar BA-30 (INA Food Industry, Nagano, Japan) before autoclaving.

### 2.2. Environmental Samples

Water, soil, and plant samples were collected from urban environments with no known history of Se contamination and used in the experiment. The samples A to L used in this study are shown in Table 1. For soil samples, 1 g of wet sample was suspended in 10 mL of sterile saline (8 g/L NaCl) and allowed to stand for 1 min, and the supernatant was used. For plants, an appropriate amount was suspended in 10 mL of sterile saline, and the supernatant was used after allowing it to stand for 1 min.

### 2.3. Enrichment and Isolation of Se-Metabolizing Microorganisms

One mL of the pretreated sample A was inoculated into TSB-MS and cultured under static conditions at 30 °C for 2 weeks. One mL of the resulting culture was inoculated into a fresh medium of the same composition and cultured under the same conditions repeatedly for 1 week to enrich for Se-metabolizing microorganisms. Se(VI) concentrations at the start and the end of each batch were analyzed, and the Se(VI) removal [%] was calculated according to Equation (1), as follows:

(*Se*(*VI*) *removal*) = ((*Se*(*VI*) *at the start*) − (*Se*(*VI*) *at the end*))/(*Se*(*VI*) *at the start*) × 100 (1)

The aliquot of the enriched culture of the seventh batch was then diluted appropriately and spread onto TSB-MS agar containing 5 mM of Se(VI).

Samples B to L were appropriately diluted and spread onto TSB agar containing 5 mM of Se(VI) without enrichment. The agar plates were cultivated at 30 °C, and red colonies, indicating the production of Se^0^ in amorphous form, were isolated. The isolated microorganisms were stored at −80 °C in 25% glycerol until use.

### 2.4. Evaluation of the Se Metabolic Potential of the Isolates

One colony of each isolated selenium-metabolizing microorganism was inoculated into TSB medium and cultured aerobically at 180 rpm for 24 h in the rotary-shaking incubator at 30 °C. In total, 200 μL of the culture was inoculated into TSB medium containing 5 mM sodium selenate and cultured anaerobically at 30 °C, 180 rpm for 1 week. An aliquot of the culture medium was taken as a sample before and after the cultivation and centrifuged (10,000× *g*, 4 °C, 15 min) to measure the total Se in the supernatant, denoted as “soluble Se” hereafter. Soluble Se removal [%] was calculated according to Equation (2), as follows:

(*Soluble Se removal*) = ((*Soluble Se at day* 0) − (*Soluble Se at day* 7))/(*Soluble Se at day* 0) × 100 (2)

### 2.5. Detailed Analysis of the Se Metabolism of the Representative Strains

One colony of the five representative strains, *Citrobacter freundii* K21-1, *Scandinavium hiltneri* K24-1, *Citrobacter braakii* K24-2, *Klebsiella aerogenes* K24-4, and *Citrobacter freundii* K24-5, was inoculated into 20 mL of TSB medium and cultivated aerobically at 30 °C and 180 rpm for 24 h using a rotary-shaking incubator, respectively. Then, 200 μL of the resulting culture was inoculated into a new medium of the same composition and cultivated under the same conditions for 24 h. In total, 200 μL of the resulting culture was inoculated into 20 mL of TSB medium containing 5 mM Se(VI) and cultivated under anaerobic conditions with rotary shaking (30 °C, 180 rpm). Individual vials were periodically sacrificed for analysis. The collected samples were centrifuged (10,000× *g*, 4 °C, 15 min) to separate the supernatant and precipitate, which were then stored at −20 °C until analysis.

### 2.6. Analytical Procedures

The Se(VI) and Se(IV) concentrations in the supernatant were analyzed using an ion chromatograph (ICS-1100, Thermo Fischer Scientific, Waltham, MA, USA). The samples were appropriately diluted with 2 mM Na_2_CO_3_, filtered using a syringe filter (DISMIC 03CP045AN, Advantec, Tokyo, Japan), and subjected to analysis. A Dionex IonPac AG12A 4 × 50 mm (Thermo Fischer Scientific) was used as the guard column, a Dionex IonPac AS12A 4 × 200 mm (Thermo Fischer Scientific) was used as the separation column, and 2 mM Na_2_CO_3_ at 1.5 mL/min was used as the eluent. Other conditions were as recommended for the Dionex IonPac AS12A. Sodium selenate (≧98%; Nacalai Tesque, Inc., Kyoto, Japan) and sodium selenite (≧97%; Nacalai Tesque) were dissolved in ultrapure water to prepare 5 mM Se(VI) and Se(IV) solutions, which were used as standard solutions.

Soluble Se was analyzed by inductively coupled plasma optical emission spectroscopy (ICP-OES; Avio200, PerkinElmer, Shelton, CT, USA) or flame atomic absorption spectrometry (FAAS; iCE3500, Thermo Fischer Scientific) with air-acetylene flame. The total Se in the precipitate, denoted as “Solid Se” hereafter, was analyzed after oxidative decomposition treatment with mixed acid (63.0% nitric acid:96.0% sulfuric acid = 20:1). In total, 4 mL of mixed acid was added to the precipitate recovered from 20 mL of culture medium by centrifugation (10,000× *g*, 4 °C, 15 min). The mixture in the centrifugation tube was tightly sealed and heated at 100 °C for 10 min. After cooling, 16 mL of ultrapure water was added and mixed, and the mixture was analyzed by ICP-OES or FAAS. A 1000 mg/L Se standard solution (Kanto Chemical, Tokyo, Japan) was used as the standard for ICP-OES and FAAS.

### 2.7. DNA Extraction and 16S rRNA Gene Sequence Analysis

Cells were harvested from 1 mL of Se-metabolizing microorganism culture by centrifugation (20,000× *g*, 4 °C, 1 min) and stored at −20 °C until use. Genomic DNA was extracted from the cells using NucleoSpin Microbial DNA (TaKaRa Bio, Shiga, Japan). The 16S ribosomal RNA gene was amplified by polymerase chain reaction using genomic DNA as a template, Tks Gflex™ DNA Polymerase (TaKaRa Bio), and primers 27F (5′-AGAGTTTGATCCTGGCTCAG-3) and 1492R (5′-GGCTACCTTGTTACGACTT-3′). The PCR products were purified using NucleoSpin Gel and PCR Clean-up (TaKaRa Bio) and entrusted to Macrogen Japan (Tokyo, Japan) for Sanger sequencing. The obtained 16S rRNA gene sequence was analyzed using EzBioCloud (https://www.ezbiocloud.net/ accessed on 16 June 2025) [15] to identify the most homologous microorganism. The multiple alignment and phylogenetic analysis of 16S rRNA gene sequences among the isolates and the type strains of the members of family Enterobacteriaceae were performed using MEGA version 12.0.11 [16].

## 3. Results and Discussion

### 3.1. Isolation of Se-Metabolizing Microorganisms

**Figure 1:**
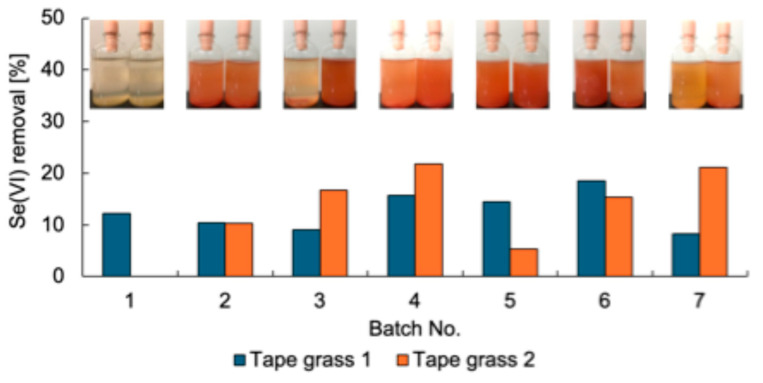
Se(VI) removal of the enrichment cultures. Inset photos are the culture after each batch. Left, Tape grass 1; right, Tape grass 2.

Se-metabolizing microorganisms were isolated from urban environmental samples A–L (Table 1). First, the enrichment of Se-metabolizing microorganisms in tape grass grown in Tomoe River, Shizuoka, Japan (sample A) was conducted. The sample was cultured repeatedly under static conditions in TSB-MS medium supplemented with 5 mM Se(VI). Duplicate enrichment cultures (designated as tape grass 1 and 2) were prepared under the same conditions. Se(VI) removal was assessed at the end of each batch culture (Figure 1). Both enrichment cultures consistently removed approximately 10–20% of the Se(VI) over seven successive batch cultures, suggesting sustained growth of Se(VI)-reducing microorganisms likely associated with the surface of the tape grass. Because these cultures were maintained under static conditions, oxygen availability was limited to diffusion from the air above the culture medium. Thus, it is plausible that the enriched microorganisms were capable of Se(VI) reduction under microaerobic or aerobic conditions. Ion chromatography analysis failed to detect Se(IV), and a light red coloration was observed in the culture fluid at the end of each batch (Figure 1), implying that any Se(IV) generated was rapidly reduced to Se^0^. The final enrichment culture was plated onto TSB-MS agar containing 5 mM Se(VI) and incubated under anaerobic conditions. A dense red colony indicative of Se^0^ production was isolated from the enrichment tape grass 2 and designated strain K21-1.

For samples B–L, appropriate pretreatment was conducted prior to direct plating onto TSB agar containing 5 mM Se(VI). From each sample, one colony displaying a distinct red coloration was selected, resulting in the isolation of strains K24-1 through K24-11.

**Figure 2:**
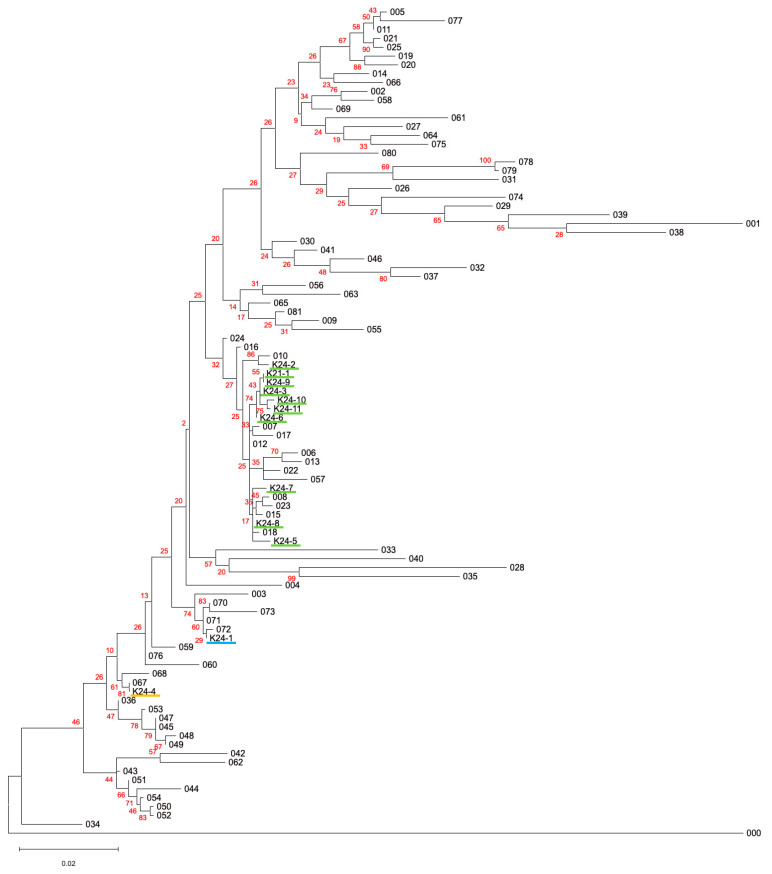
Phylogenetic tree of 16S rRNA gene sequences among the 12 isolates and the type strains of the members of the family Enterobacteriaceae. Numbers 001–081 are the type strains of the members of the family Enterobacteriaceae. Number 000 is the outgroup (*Pseudomonas aeruginosa*). The colors of the underbars of the 12 isolates correspond to the taxon: light green, *Citrobacter* spp.; light blue, *Scandinavium hiltneri*; yellow, *Klebsiella aerogenes*. The red numbers indicate the bootstrap values in 100 replicates. Detailed information is available in Table S1.

All 12 isolates were subjected to 16S rRNA gene sequencing (Table 2). The phylogenetic tree of 16S rRNA gene sequences among the 12 isolates and the type strains of the members of the family Enterobacteriaceae is shown in Figure 2. Sequence homology analyses revealed that all isolates were closely related to type strains belonging to the family Enterobacteriaceae, which may reflect that the culture media used in this study (TSB supplemented with 5 mM Se(VI)) specifically determined the growth of the members of the Enterobacteriaceae. Strains K24-1, K24-2, and K24-4 showed >99% sequence identity with type strains of *Scandinavium hiltneri*, *Citrobacter braakii*, and *Klebsiella aerogenes*, respectively. The remaining nine isolates were identified as *Citrobacter freundii*.

### 3.2. Evaluation of the Se Metabolic Potential in Isolates

**Figure 3:**
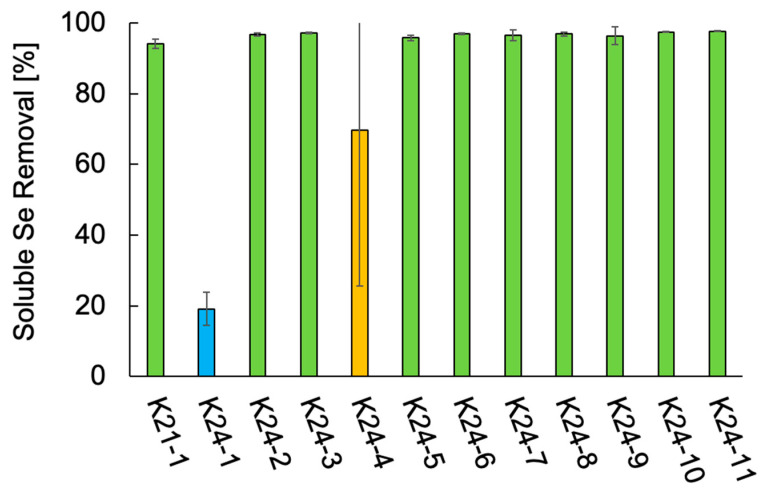
Soluble Se removal of the 12 isolates. Error bars represent the standard deviation of three independent experiments. Light green, *Citrobacter freundii* and *Citrobacter braakii*; light blue, *Scandinavium hiltneri*; yellow, *Klebsiella aerogenes*.

The Se metabolic capabilities of the 12 isolates were assessed by comparing their ability to reduce soluble Se species. The soluble Se removal of the 12 isolates is shown in Figure 3. All isolates exhibited some level of Se removal. Ten isolates, all identified as *Citrobacter* spp. (K21-1, K24-2, K24-3, K24-5 through K24-11), removed over 95% of the supplemented Se. In contrast, *S. hiltneri* K24-1 demonstrated the lowest removal efficiency (19%). Two cultures of *K. aerogenes* K24-4 exhibited removal rates exceeding 90%, although that of the other culture was 19%, resulting in 69% on average. These results confirm that all 12 isolates possess Se metabolic capabilities. In particular, the *Citrobacter* strains demonstrated high Se removal efficiency, suggesting robust Se-metabolizing activity.

### 3.3. Se Metabolism in Selected Strains: K21-1, K24-1, K24-2, K24-4, and K24-5

**Figure 4:**
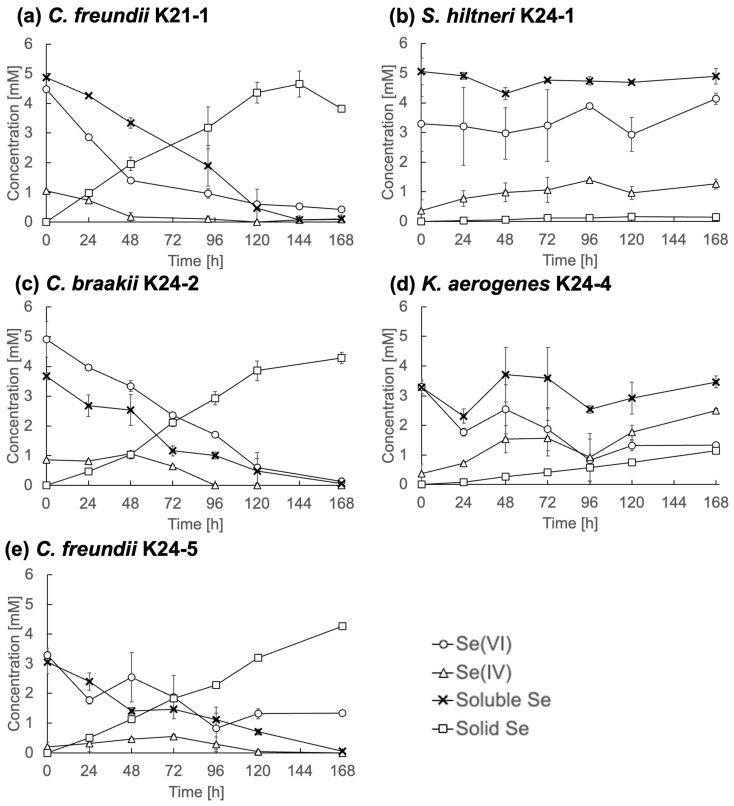
Se metabolism in selected strains. (**a**) *Citrobacter freundii* K21-1; (**b**) *Scandinavium hiltneri* K24-1; (**c**) *C. braakii* K24-2; (**d**) *Klebsiella aerogenes* K24-4; (**e**) and *C. freundii* K24-5. Error bars represent the standard deviation of three independent experiments.

To further characterize Se metabolism, five representative strains, *C. freundii* K21-1, *S. hiltneri* K24-1, *C. braakii* K24-2, *K. aerogenes* K24-4, and *C. freundii* K24-5, were analyzed in detail. Each was cultured in TSB medium containing 5 mM Se(VI), and changes in Se speciation were monitored (Figure 4).

*C. freundii* K21-1, *C. braakii* K24-2, and *C. freundii* K24-5 (Figure 4a, Figure 4c, and Figure 4e, respectively) displayed similar Se reduction profiles. Se(VI) concentrations declined steadily and fell below 0.5 mM by the end of the cultivation period. Se(IV) concentrations remained consistently below 1 mM, while solid Se accumulated over time. These observations indicate efficient reduction of Se(IV) to Se^0^, without significant intermediate accumulation. This contrasts with many previously described Se(VI)-reducing bacteria, which often display limited Se(IV)-reducing activity [9,17]. The findings suggest that these *Citrobacter* strains are highly efficient Se reducers. The rapid reduction of Se(IV) under anaerobic conditions implies the presence of an active reduction mechanism, e.g., anaerobic respiration or detoxification, in the isolates. Molecular biological analyses are required for a deeper understanding.

In contrast, *S. hiltneri* K24-1 showed an increase in Se(IV) concentration to 1.3 mM by the end of cultivation (Figure 4b), with no appreciable reduction in overall soluble Se or increase in solid Se. Notably, the initial Se(VI) concentration was 3.3 mM, lower than the supplemented 5 mM, indicating rapid adsorption or partial uptake. These results suggest limited Se metabolic activity, despite the ability to reduce Se(VI) to Se(IV).

*K. aerogenes* K24-4 reduced Se(VI) to 1.3 mM, produced 2.5 mM Se(IV), and generated 1.1 mM solid Se (Figure 4d). This profile indicates Se metabolic activity, although with limited efficiency under the tested conditions.

*Citrobacter* spp. are widespread in aquatic ecosystems, and their involvement in Se cycling is well documented. For instance, *C. freundii* Iso Z7 was isolated from a Se-impacted lake sediment, and its efficient Se(VI) to Se^0^ reduction was demonstrated [18]. *C. braakii* was isolated from a Se-remediation facility, where its Se-removal capacity was enhanced by zero-valent iron [19]. The Se metabolism in *Citrobacter* sp. JSA isolated from freshwater sediment was characterized [20]. Se(IV) reduction by *Citrobacter* sp. NVK-2 isolated from activated sludge was significantly enhanced when the lactate was periodically supplemented with the culture [21]. The genome analysis of *C. freundii* RLS1 predicted the involvement of the *ynfEGH* in Se(VI) reduction [22].

While the reduction of Se(VI) to Se^0^ by *Citrobacter* spp. is well-established, the simultaneous isolation of multiple Se(VI)-reducing *Citrobacter* strains from diverse urban environments is notable. This contrasts with previous research, which has predominantly focused on Se-metabolizing bacteria from contaminated habitats [6,7,8,9,12,13,14]. The widespread presence of such organisms in urban environments has implications for understanding natural Se biogeochemistry. It remains unclear whether Se metabolism is a universal trait within *Citrobacter* spp. However, Theisen and Yee (2014) noted that the putative Se(VI) reductase operon *ynfEGH* is conserved across the genus [22], warranting further molecular ecological studies to elucidate its distribution and functional significance.

Additionally, this study provides the first indication of Se metabolism in *S. hiltneri* under laboratory conditions. *S. hiltneri*, a newly described species within Enterobacteriaceae [23], has not been previously associated with Se transformation. *Klebsiella* strains from river sediments also demonstrated Se reduction, although at lower efficiencies [24]. These observations suggest that, despite relatively lower activity under artificial conditions, these species may affect the fate of Se in natural ecosystems.

## 4. Conclusions

To gain a deeper understanding of Se-metabolizing microorganisms, which play a key role as mediators in the biogeochemical cycling of Se, this study elucidated the Se metabolism of 12 bacterial strains isolated from urban environmental samples. Notably, 10 of these strains, identified as members of the genus *Citrobacter*, demonstrated the ability to rapidly reduce high concentrations of Se(VI). Despite originating from environments not contaminated with Se, these bacteria exhibited pronounced Se-metabolizing activity, suggesting that such metabolic capacity is an inherent characteristic of *Citrobacter* sp. Consequently, the presence of *Citrobacter* may indicate Se metabolic potential in environmental microbial communities. Future research should clarify the molecular biological mechanisms underlying Se metabolism in *Citrobacter*, thereby facilitating a more detailed understanding of the ecology of Se-metabolizing microorganisms in natural environments.

## Figures and Tables

**Table 1:** Environmental samples used in this study.

	Sample	Type	Location
A	tape grass	plant	Tomoe River, Shizuoka, Japan
B	vegetable field	soil	Private house A, Shizuoka, Japan
C	flower bed	soil	Private house A, Shizuoka A, Japan
D	hibiscus	plant	Private house B, Shizuoka, Japan
E	garden 1	soil	Private house B, Shizuoka, Japan
F	garden 2	soil	Private house C, Shizuoka, Japan
G	drainage ditch	water	Private house C, Shizuoka, Japan
H	green foxtail 1	plant	Roadside, Shizuoka Japan
I	green foxtail 2	plant	Roadside, Shizuoka Japan
J	moss 1	plant	Shrine A, Shizuoka Japan
K	moss 2	plant	Shrine B, Shizuoka Japan
L	seashore	water	Sea front park, Shizuoka, Japan

**Table 2:** 16S rRNA gene sequence homology of isolated strains.

Strain	Source	EZBioCloud_Tophit				
		Taxon	Strain	Accession No.	Similarity [%]	Completeness [%]
K21-1	A	*Citrobacter freundii*	DSM30039	AJ233408	99.64	96
K24-1	B	*Scandinavium hiltneri*	H11S7	OM987267	99.72	96
K24-2	C	*Citrobacter braakii*	ATCC51113	NAEW01000064	99.57	94.7
K24-3	D	*Citrobacter freundii*	DSM30039	AJ233408	99.57	96
K24-4	E	*Klebsiella aerogenes*	KCTC2190	CP002824	99.79	96
K24-5	F	*Citrobacter freundii*	DSM30039	AJ233408	99.36	96
K24-6	G	*Citrobacter freundii*	DSM30039	AJ233408	99.65	96.2
K24-7	H	*Citrobacter freundii*	DSM30039	AJ233408	99.72	96.5
K24-8	I	*Citrobacter freundii*	DSM30039	AJ233408	99.72	96.1
K24-9	J	*Citrobacter freundii*	DSM30039	AJ233408	99.64	96
K24-10	K	*Citrobacter freundii*	DSM30039	AJ233408	99.43	96.5
K24-11	L	*Citrobacter freundii*	DSM30039	AJ233408	99.36	95.4

## Data Availability

The original data presented in the study are openly available in the Tokoha University Repository at https://doi.org/10.18894/0002000495. The 16S rRNA gene sequences were deposited in the DNA Data Bank of Japan (DDBJ) under the accession nos. LC882276 to LC882287.

## Supplementary Materials

The following supporting information can be downloaded at: https://www.mdpi.com/article/10.3390/microorganisms13071675/s1, Table S1: List of the bacterial strains and accession numbers of 16S rRNA gene used in the phylogenetic tree (Figure 2).

## Abbreviations

The following abbreviations are used in this manuscript:

Se(VI)	Selenate
Se(IV)	Selenite
Se^0^	Elemental selenium
ICP-OES	Inductively coupled plasma optical emission spectrometry
FAAS	Flame atomic absorption spectrometry
