# Supplementary material for: Selenium Metabolizing Capabilities of 12 Bacterial Strains Isolated from Urban Environmental Samples
Source: Microorganisms. 2025 Jul 16;13(7):1675. doi: 10.3390/microorganisms13071675 (PMC12298126; doi:10.3390/microorganisms13071675)
Supplement: Supplementary file 1 [file microorganisms-13-01675-s001.zip › microorganisms-3731825-supplementary.pdf]

Supplementary Material
Selenium metabolizing capabilities of the twelve bacterial strains isolated from urban environmental samples
Masashi Kuroda, Iori Ishimoto, Chisato Kameoka, Toshiki Kawanishi, Ren Saito, Hajime Toki, Hiroya Yamagishi, Yuzuki Watanabe and Yukinori Tani

Table S1. List of the bacterial strains and accession numbers of 16S rRNA gene used in the phylogenetic tree (Figure 2) (1/3)

No.	Taxon name	strain	Accesion No
0	Pseudomonas aeruginosa	JCM 5962(T)	BAMA01000316
1	Affinibrenneria salicis	L3-3HA(T)	MN036523
2	Atlantibacter hermannii	FDAARGOS_888(T)	CP065701
3	Buttiauxella agrestis	ATCC 33320(T)	JMPI01000079
4	Cedecea davisae	DSM 4568(T)	ATDT01000040
5	Citrobacter amalonaticus	CECT 863(T)	FR870441
6	Citrobacter arsenatis	LY-1(T)	MK262983
7	Citrobacter braakii	ATCC 51113(T)	NAEW01000064
8	Citrobacter cronae	Tue2_1(T)	MN548424
9	Citrobacter enshiensis	S171(T)	OP183215
10	Citrobacter europaeus	97/99(T)	FLYB01000015
11	Citrobacter farmeri	CDC 2991-81(T)	AF025371
12	Citrobacter freundii	DSM 30039(T)	AJ233408
13	Citrobacter gillenii	CDC 4693-86(T)	AF025367
14	Citrobacter koseri	LMG 5519(T)	HQ992945
15	Citrobacter meridianamericanus	BR102(T)	ON890426
16	Citrobacter murliniae	CDC 2970-59(T)	AF025369
17	Citrobacter pasteurii	CIP 55.13(T)	CDHL01000036
18	Citrobacter portucalensis	A60(T)	MVFY01000035
19	Citrobacter rodentium	NBRC 105723(T)	BBNA01000105
20	Citrobacter sedlakii	NBRC 105722(T)	BBNB01000023
21	Citrobacter telavivensis	6105(T)	MN603664
22	Citrobacter tructae	SNU WT2(T)	MN093886
23	Citrobacter werkmanii	NBRC 105721(T)	BBMW01000025
24	Citrobacter youngae	CCUG 30791(T)	RPOI01000045
25	CP011132_s	Y19	CP011132
26	Cronobacter sakazakii	NBRC 102416(T)	BAWU01000071
27	Dryocola boscaweniae	H6W4(T)	OM971056
28	EF633999_s	M-5	EF633999
29	Enterobacillus tribolii	DSM 103736(T)	QRAP01000026
30	Enterobacter cloacae subsp. Cloacae	ATCC 13047(T)	CP001918

Table S1. List of the bacterial strains and accession numbers of 16S rRNA gene used in the phylogenetic tree (Figure 2), continued (2/3)

No.	Taxon name	strain	Accesion No
31	Entomohabitans teleogrylli	SCU-B244(T)	KJ000798
32	Escherichia coli	ATCC 11775(T)	X80725
33	EU430753_s	ZJUPD3	EU430753
34	Franconibacter helveticus	513/05(T)	DQ273688
35	HM640295_s	YUST-DW17	HM640295
36	Huaxiibacter chinensis	155047(T)	OL712205
37	Intestinirhabdus alba	BIT-B35(T)	MK734184
38	Izhakiella capsodis	N6PO6(T)	KF436763
39	Jejubacter calystegiae	KSNA2(T)	MK931324
40	JN975122_s	b26	JN975122
41	CP046115_s	P620	CP046115
42	Enterobacter lignolyticus	SCF1(T)	CP002272
43	Klebsiella africana	Kp7(T)	MK040622
44	Klebsiella granulomatis	KH 22	AF010251
45	Klebsiella grimontii	06D021(T)	FZTC01000044
46	Klebsiella indica	TOUT106(T)	MK942857
47	Klebsiella michiganensis	W14(T)	JQ070300
48	Klebsiella oxytoca	JCM 1665(T)	AB004754
49	Klebsiella pasteurii	SPARK_836_C1(T)	MN091366
50	Klebsiella pneumoniae subsp. Pneumoniae	DSM 30104(T)	AJJI01000018
51	Klebsiella quasipneumoniae subsp. Quasipn	01A030(T)	HG933296
52	Klebsiella quasivariicola	KPN1705(T)	CP022823
53	Klebsiella spallanzanii	SPARK_775_C1(T)	MN091365
54	Klebsiella variicola subsp. Variicola	DSM 15968(T)	CP010523
55	LGIT_s	RIT-PI-d	LGIT01000014
56	QBJD_s	RIT 418	QBJD02000037
57	Kluyvera ascorbata	ATCC 33433(T)	JMPL01000225
58	Kosakonia cowanii	JCM 10956(T)	BBEU01000098
59	Leclercia adecarboxylata	NBRC 102595(T)	BCNP01000062
60	Lelliottia nimipressuralis	LMG 10245(T)	Z96077
61	Mangrovibacter plantisponsor	MSSRF40(T)	EF643377
62	Phytobacter diazotrophicus	LS 8(T)	DQ821583
63	Pluralibacter gergoviae	JCM 1234(T)	AB004748
64	PQLZ_s	S648	PQLZ01000008
65	Pseudescherichia vulneris	NBRC 102420(T)	BBMZ01000044
66	Pseudocitrobacter faecalis	DSM 27453(T)	QNRL01000033
67	Klebsiella aerogenes	KCTC 2190(T)	CP002824

Table S1. List of the bacterial strains and accession numbers of 16S rRNA gene used in the phylogenetic tree (Figure 2), continued (3/3)

No.	Taxon name	strain	Accesion No
68	Raoultella planticola	ATCC 33531(T)	JMPP01000074
69	Salmonella enterica subsp. Enterica	LT2(T)	AE006468
70	Scandinavium goeteborgense	CCUG 66741(T)	MK558235
71	Scandinavium hiltneri	H11S7(T)	OM987267
72	Scandinavium manionii	H17S15(T)	OM987268
73	Scandinavium tedordense	TWS1a(T)	OM987269
74	Shimwellia pseudoproteus	521(T)	FJ267523
75	Siccibacter turicensis	z508(T)	AVPP01000089
76	Silvania hatchlandensis	H19S6(T)	OM987253
77	Superficieibacter electus	BP-1(T)	PQGD01000069
78	Tenebrionibacter intestinalis	BIT-L3(T)	MW411192
79	Tenebrionicola larvae	YMB-R21(T)	MW680835
80	Trabulsiella guamensis	ATCC 49490(T)	JMTB01000142
81	Yokenella regensburgei	ATCC 49455(T)	JMPS01000045
82		K21-1	LC882276
83		K24-1	LC882277
84		K24-2	LC882278
85		K24-3	LC882279
86		K24-4	LC882280
87		K24-5	LC882281
88		K24-6	LC882282
89		K24-7	LC882283
90		K24-8	LC882284
91		K24-9	LC882285
92		K24-10	LC882286
93		K24-11	LC882287
